# Involvement of Intracellular TAGE and the TAGE–RAGE–ROS Axis in the Onset and Progression of NAFLD/NASH

**DOI:** 10.3390/antiox12030748

**Published:** 2023-03-19

**Authors:** Akiko Sakasai-Sakai, Kenji Takeda, Masayoshi Takeuchi

**Affiliations:** Department of Advanced Medicine, Medical Research Institute, Kanazawa Medical University, 1-1 Daigaku, Uchinada-machi, Ishikawa 920-0293, Japan

**Keywords:** advanced glycation end-products (AGEs), toxic AGEs (TAGE), receptor for AGEs (RAGE), nonalcoholic fatty liver disease (NAFLD)/nonalcoholic steatohepatitis (NASH), hepatocellular carcinoma (HCC)

## Abstract

The repeated excessive intake of sugar, a factor that contributes to the onset of nonalcoholic fatty liver disease (NAFLD) and its progression to the chronic form of nonalcoholic steatohepatitis (NASH), markedly increases the hepatocyte content of glyceraldehyde (GA), a glucose/fructose metabolic intermediate. Toxic advanced glycation end-products (toxic AGEs, TAGE) are synthesized by cross-linking reactions between the aldehyde group of GA and the amino group of proteins, and their accumulation has been implicated in the development of NAFLD/NASH and hepatocellular carcinoma (HCC). Our previous findings not only showed that hepatocyte disorders were induced by the intracellular accumulation of TAGE, but they also indicated that extracellular leakage resulted in elevated TAGE concentrations in circulating fluids. Interactions between extracellular TAGE and receptor for AGEs (RAGE) affect intracellular signaling and reactive oxygen species (ROS) production, which may, in turn, contribute to the pathological changes observed in NAFLD/NASH. RAGE plays a role in the effects of the extracellular leakage of TAGE on the surrounding cells, which ultimately promote the onset and progression of NAFLD/NASH. This review describes the relationships between intracellular TAGE levels and hepatocyte and hepatic stellate cell (HSC) damage as well as the TAGE–RAGE–ROS axis in hepatocytes, HSC, and HCC cells. The “TAGE theory” will provide novel insights for future research on NAFLD/NASH.

## 1. Introduction

Nonalcoholic fatty liver disease (NAFLD) covers of a broad spectrum of chronic liver diseases (CLD) ranging from nonalcoholic fatty liver (NAFL) and nonalcoholic steatohepatitis (NASH) to cirrhosis and/or hepatocellular carcinoma (HCC). NAFLD is a worldwide health issue and potentially the most important CLD in the developed world in the 21st century [1]. The number of patients with NAFLD has been rapidly increasing, and is estimated to affect between 25 and 30% of the general adult population worldwide [2,3]. Approximately 3 to 6% of NAFLD patients may also have NASH as an underlying disease [4,5]. NAFLD has also been implicated in other medical conditions, such as insulin resistance (IR), obesity, metabolic syndrome (MS), cardiovascular disease (CVD), and type 2 diabetes mellitus (T2DM). Numerous studies, including extensive reviews, have confirmed the progression of NALFD through the stages of steatosis, ballooning, inflammation, fibrosis, cirrhosis, and HCC [1,6]. However, the pathogenesis of the sequential stages of NAFLD remains unclear.

Hyperglycemia initiates and contributes to the progression of a non-enzymatic glycation reaction between proteins that results in the production of advanced glycation end-products (AGEs), the types of which are dependent on the reducing sugars or carbonyl compounds involved in the reaction [7,8]. AGEs formed from glyceraldehyde (GA), a fructose (Fru)/glucose (Glu) metabolic intermediate, are highly cytotoxic and, thus, are called toxic AGEs (TAGE). Our recent findings showed that intracellular TAGE damaged not only liver cells [9,10,11,12], but also other cell types, including neuroblasts, pancreatic cancer cells, cardiomyocytes, myoblasts, pancreatic islet β-cells, osteoblasts, and cardiac fibroblasts [13,14,15,16,17,18,19]. The leakage of cytotoxic intracellular TAGE into the blood has been suggested to increase their concentration in circulating fluids [20,21,22]. Extracellular TAGE and receptor for AGEs (RAGE) interactions were found to affect intracellular signaling, gene expression, and the release of pro-inflammatory molecules and promoted reactive oxygen species (ROS) production in many cell types [23,24,25,26,27], and these changes collectively contribute to the development of NASH.

Processed foods with a high content of sugar (Fru, high-Fru corn syrup (HFCS), and sucrose), sugar-sweetened beverages (SSB) [28] and/or dietary AGEs (mainly Glu-/Fru-AGEs [29]), when consumed repeatedly and at excessive amounts, have been implicated in the development of obesity and MS as well as the onset and progression of NAFLD/NASH, T2DM, and CVD; however, the mechanisms responsible have not yet been elucidated [30,31,32,33,34]. A range of “parallel hits”, such as nutritional factors, environmental elements, IR, and oxidative stress, play a role in the progression of NAFL to NASH [35]. The chronic excessive intake of sugars has been shown to affect hepatic metabolism, resulting in high GA levels, which leads to the production of and increases in TAGE [20,21,27].

This review describes the relationships between the intracellular production and accumulation of TAGE and hepatocyte damage as well as the TAGE–RAGE–ROS axis in hepatocytes, hepatic stellate cells (HSC), and HCC cells.

## 2. Production of AGEs in the Human Body

**Figure 1:**
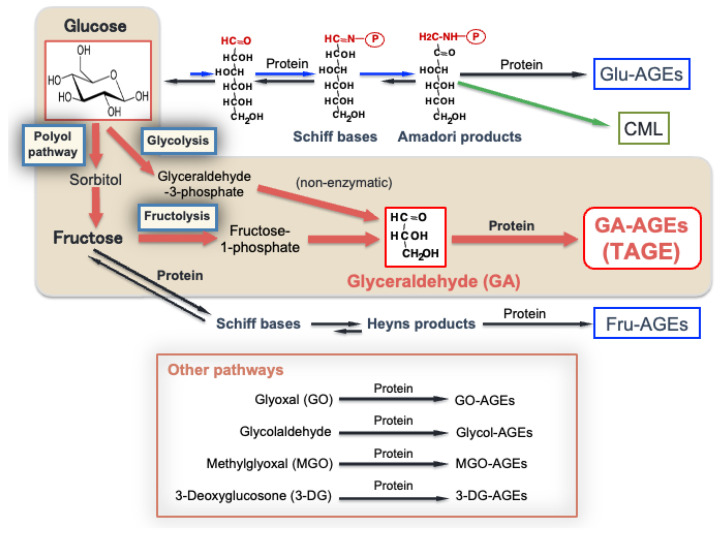
Numerous pathways for the in vivo generation of AGEs.

Seven different classes of AGEs and N^ε^-(carboxymethyl)lysine (CML) have been identified in human serum [36,37,38,39] (Figure 1). Since GA-derived AGEs are highly cytotoxic [40], we proposed the novel concept of TAGE [41]. We prepared a specific anti-TAGE antibody that bound to epitopes to GA-derived structures, but not to AGEs with well-characterized CML and N^ε^-(carboxyethyl)lysine [37]. In addition to these structures, the anti-TAGE antibody did not recognize various AGEs produced from reducing sugars and carbonyl molecules, such as pentosidine, pyrraline, argpyrimidine, crossline, and glyoxal (GO)-/methylglyoxal (MGO)-lysine dimers [37,42]. Although the anti-TAGE antibody did not bind to AGEs with structures that had not been elucidated, including Glu-/GA-/Fru-/GO-/glycolaldehyde-/MGO-/3-deoxyglucosone-derived AGEs [37,42], its binding to different epitopes to GA-AGE-derived structures, such as GLAP (a 3-hydroxy-5-hydroxymethyl-pyridinium compound) [43], triosidines [44], and MG-H1 (a MGO-derived hydroimidazolone compound) [45]). None of these structures showed AGE-specific fluorescence or protein cross-linking. Two compounds with a 1,4-dihydropyrazine ring that showed fluorescence and had cross-links were identified as TAGE candidate structures [PCT/JP2019/34195].

We previously demonstrated that serum AGE fractions from patients with diabetic nephropathy with hemodialysis (DN-HD) were neurotoxic and that the anti-TAGE antibody neutralized these effects [40,46]. Therefore, among GA-derived AGE structures, only those to which the anti-TAGE antibody binds appear to be cytotoxic. Accordingly, AGEs that bind the anti-TAGE antibody were referred to as toxic AGEs (TAGE) [47,48].

The amino/guanidino groups of proteins and reducing sugars (glyceraldehyde (GA), glucose, and fructose) react in a non-enzymatic manner and produce reversible Schiff bases and Amadori products/Heyns products. AGEs, heterogeneous fluorescent derivatives, are the products of the irreversible cross-linking of these early glycation products through further rearrangements, dehydration, condensation reactions, and other complex reactions. Glu-AGEs: glucose-derived AGEs; CML: N^ε^-(carboxymethyl)lysine; GA-AGEs: glyceraldehyde-derived AGEs; TAGE: toxic AGEs; Fru-AGEs: fructose-derived AGEs; GO-AGEs: glyoxal-derived AGEs; Glycol-AGEs: glycolaldehyde-derived AGEs; MGO-AGEs: methylglyoxal-derived AGEs; 3-DG-AGEs: 3-deoxyglucosone-derived AGEs.

## 3. The Intracellular Generation and Accumulation of TAGE in the Liver

Cells produce GA through three pathways [49]: (i) glycolysis, (ii) fructolysis, and (iii) the polyol pathway, which increases the production of TAGE by intracellular proteins. (i) Glycolysis plays an important role in Glu metabolism. GA-3-P dehydrogenase (GAPDH) metabolizes GA-3-phosphate (GA-3-P), an intermediate of this pathway. GA-3-P accumulates with a decrease in GAPDH activity and, thus, is shifted to another metabolic pathway for its non-enzymatic dephosphorylation to GA [50]. (ii) Fructolysis is crucial for Fru metabolism, particularly in the liver. Fructokinase rapidly phosphorylates Fru to Fru-1-phosphate, which is cleaved by aldolase B to GA and dihydroxyacetone phosphate (DHAP) [51]. (iii) Under hyperglycemic states in tissues that do not depend directly on insulin to absorb glucose, including the liver, brain, and heart, aldose reductase catalyzes the conversion of Glu to sorbitol in the polyol pathway, and sorbitol is then converted to Fru by sorbitol dehydrogenase [50]. Fru is subsequently metabolized by the fructolytic pathway to GA.

MGO is mainly produced as a byproduct of glycolysis via the non-enzymatic degradation of GA-3-P and DHAP, the intermediate products of glycolysis. Glycolysis contributes approximately 90% of the total amount of MGO, with the remaining 10% being generated from the auto-oxidation of Glu and degradation of glycated proteins (Amadori products) [52] and dysregulated lipid metabolism associated with the hepatic accumulation of triglycerides [53]. Previous studies reported elevated levels of MGO-AGEs in patients with liver disease [54]. Serum MGO-AGEs and soluble form of RAGE (sRAGE) levels in non-DM patients are associated with NAFLD. In addition, liver steatosis and inflammation have been shown to increase serum levels of MGO-AGEs.

We recently demonstrated that the excess consumption of 10% HFCS 55 (Fru 55%/Glu 45%) increased intracellular TAGE levels in normal rat livers and serum TAGE levels [55,56]. The HFCS-induced promotion of TAGE generation in normal rat livers may increase the risk of NAFLD/NASH. Furthermore, TAGE-induced cell death may increase the cellular leakage of TAGE, which then interact with RAGE on the surrounding cells. Therefore, intracellular TAGE may spread throughout the body.

## 4. The TAGE–RAGE Axis

Receptor-dependent/-independent mechanisms have been shown to play a role in cellular dysfunction and tissue damage caused by AGEs. A previous study examined different classes of biological reactions mediated by RAGE and identified a number of AGE-binding proteins [48]. RAGE is a multiligand cell surface protein that functions as a pattern recognition receptor and is a member of the immunoglobulin superfamily, the characteristics of which have been intensively investigated [57,58,59]. RAGE is present within the plasma membrane in an oligomeric or preassembled state [60,61,62]. The majority of healthy adult tissues express low levels of RAGE; however, its expression is up-regulated under pathological conditions, such as T2DM, CVD, and cancer [23,62,63].

Previous studies reported that TAGE and RAGE interactions activated the transcription factors NF-κB/AP-1 and Rac-1/cdc42, which, in turn, induced cellular responses through distinct pathways, thereby producing cytokines and triggering cell migration [59]. RAGE is expressed by different cell types, such as hepatocytes, HSC, endothelial cells (EC), and pericytes [25]. Previous studies demonstrated the contribution of the RAGE signaling pathway to the onset and progression of many types of liver injury [64,65]. The binding of RAGE and TAGE was shown to induce the generation of oxidative stress followed by inflammatory responses in various cell types, including hepatocytes and HSC [64].

We previously investigated the binding affinity of AGEs to RAGE. We conducted in vitro experiments to examine the binding of the seven distinct classes of AGEs and CML, which were identified in blood collected from DN-HD patients [36,37,38,39], to RAGE using a purified human RAGE protein, and found that the dissociation constant for TAGE was 0.36 μM. Similar binding kinetics were noted in cellular assays using COS-7, a fibroblast-like cell line expressing RAGE [66,67]. The following in vivo roles of TAGE and RAGE were demonstrated: (i) the anti-TAGE antibody neutralized the neurotoxic effects of various AGE structures in a serum fraction collected from DN-HD patients [40]; (ii) the neurotoxicity of AGE-rich serum collected from DN-HD patients against EC was also exhibited by TAGE [68].

The strong binding of TAGE to RAGE has been shown to induce a number of intracellular effects. Previous studies in many cell types reported that the TAGE–RAGE axis promoted the production of ROS through the activation of nicotinamide adenine dinucleotide phosphate-reduced oxidase (NOX), the expression of vascular endothelial growth factor (VEGF), and the generation of inflammatory cytokines [24,25,69,70], and thus, has been implicated in not only the onset, but also the progression of NAFLD/NASH. The TAGE–RAGE axis may also play a role in diabetic retinopathy as well as NAFLD/NASH. AGEs have been shown to increase vascular permeability via the phosphorylation of vascular endothelial (VE)-cadherin, an adhesion complex protein in adherens junctions, following the phosphorylation of Src, a tyrosine kinase, by ROS produced via NOX [71]. We recently demonstrated that TAGE disrupted adherens junctions and tight junctions and increased vascular permeability in EC [72]. Ras guanyl nucleotide-releasing protein 2 was found to protect against the TAGE-induced disruption of VE-cadherin, suggesting its potential in the development of novel therapies for TAGE-induced diabetic retinopathy [72].

As AGE receptors, AGE-R1 (OST-48)/-R2 (80K-H)/-R3 and the scavenger receptor family (classes A, B, E, and H), among others, and stabilin-1/-2, which have the opposite function to RAGE, are essential for maintaining AGE homeostasis [25,48]. Previous studies demonstrated that the expression of AGE receptors fluctuated among different cell and tissue types as a result of metabolic changes [73,74]. However, it currently remains unclear whether TAGE in addition to RAGE bind to these receptors and if AGE receptors inhibit the toxicity of TAGE.

## 5. Serum TAGE Levels and NASH/Non-B, Non-C (NBNC)-HCC

NAFLD covers a broad spectrum of diseases from NAFL to NASH. Since an excessive intake of sugar is considered to be one of the factors contributing to the onset of NASH, the relationship between TAGE and NASH has been investigated. AGEs, such as Glu-AGEs, CML, and TAGE, were measured in the sera of 66 patients with histologically defined NASH without cirrhosis, 10 with NAFL, and 30 control subjects [75]. The levels of TAGE in the sera of NASH patients were significantly higher than those in the sera of NAFL patients and healthy controls. Serum TAGE levels were also examined as a marker to discriminate between NAFL and NASH. Receiver operating characteristic curves for serum TAGE showed a threshold of 8.53 U/mL for predicting NASH. At this threshold, sensitivity and specificity were 66.7 and 88.9%, respectively. Furthermore, serum TAGE levels positively correlated with the homeostasis model assessment of IR (HOMA-IR) and inversely correlated with adiponectin levels. However, no significant differences were observed in Glu-AGEs or CML levels among the above groups. TAGE were also detected in the hepatocytes of patients with NASH, but were negligible in those with NAFL. These findings were not obtained using Glu-AGEs or CML [75].

Elevated TAGE levels in NASH and variations in TAGE levels with the attenuation of NASH are important for their superiority as a biomarker. We previously reported decreases in serum TAGE levels in 43 biopsy-proven NASH patients with dyslipidemia treated with the HMGA-CoA reductase inhibitor atorvastatin [76]. The activities of γ-glutamyl transpeptidase (γ-GTP) and alanine aminotransferase significantly decreased in all patients treated for six months with atorvastatin at 10 mg/day. Furthermore, these patients had significantly lower serum TAGE levels, with increased plasma adiponectin levels and decreased tumor necrosis factor-α (TNF-α) levels in NASH and NAFL patients.

TAGE may also be used as a biomarker to differentiate between NBNC-HCC and NASH. TAGE levels in the sera of NBNC-HCC patients were significantly higher than those in the sera of NASH patients without HCC and control subjects [77]. Serum TAGE levels (inversely) independently correlated with age, γ-GTP levels, and high-density lipoprotein cholesterol levels in a multiple stepwise regression analysis.

We also demonstrated that increases in the serum levels of TAGE positively correlated with sRAGE levels in non-DM/DM subjects, which may reflect the expression of RAGE in tissues [78,79,80,81,82].

Collectively, these findings suggest the potential of serum TAGE levels as a biomarker of NASH progression and TAGE–RAGE axis activation.

## 6. Cytotoxicity of TAGE in the Liver

SSB and processed foods with a high sugar content (i.e., HFCS and sucrose) [28], when consumed repeatedly and at excessive amounts, affect hepatic metabolism and promote the production of GA, which ultimately leads to the formation of TAGE. Based on their intracellular and extracellular cytotoxic properties, TAGE have been suggested to contribute to not only the onset, but also the progression of NASH [9,10,11,12,25,26,69,70,83,84,85]. The cytotoxicity of TAGE in hepatocytes and the role of HSC in the onset and progression of NASH are discussed below.

### 6.1. Intracellular TAGE and Hepatocyte Cell Death

Hepatocyte cell death is one of the hallmarks of NASH. Since a more detailed understanding of the causes of hepatocyte cell death will provide insights into the pathogenesis of NASH, the relationship between the accumulation of TAGE and cell death is being examined. Cell death correlated with the accumulation of TAGE in HCC cell lines (HepG2 and Hep3B), hepatocyte-like cells (HLC) differentiated from human induced pluripotent stem cells (hiPSC) (hiPSC-HLC), and primary cultured hepatocytes [9,10,11,12]. Moreover, necrosis-like cell death occurred in HepG2 cells in which TAGE had accumulated, which, in turn, appeared to elicit massive inflammatory responses in the surrounding cells [10]. Necrosis-like cell death has been shown to occur at sites of NASH disease [86], suggesting that one of the causes of necrosis-like cell death is the accumulation of TAGE.

### 6.2. TAGE-Modified Proteins and Hepatocyte Cell Damage

The accumulation of TAGE in hepatocytes correlates with cell death, and functional abnormalities as a result of TAGE modifications to various proteins may be expected in the cell death process. Proteins that are crucial for the maintenance of cellular functions are subject to TAGE modifications.

(i) TAGE modifications and a decrease in the chaperone activity of the heat shock cognate 70 (Hsc70) protein without changes to its mRNA expression level were detected in GA-treated Hep3B cells [9]. Intracellular protein homeostasis is maintained by Hsc70 through its regulation of protein folding, transport, and degradation [87]. The loss of the chaperone activity of TAGE-modified Hsc70 indicated a lack of intracellular protein quality control.

(ii) Previous studies demonstrated that TAGE targeted the RNA-binding protein, heterogeneous nuclear ribonucleoprotein M (hnRNPM) in Hep3B cells cultured in media containing GA or a high Fru content [83,84]. hnRNPM is involved in a number of processes in the metabolism of nucleic acids, such as alternative splicing, the stabilization of mRNA, and regulation of transcription and translation. Furthermore, the knockdown of hnRNPM was found to up- or down-regulate the expression of genes associated with extracellular exosome-containing extracellular spaces [84]. Due to its potential as a biomarker of NASH, further studies are needed on the role of hnRNPM in the extracellular space.

(iii) Caspase-3 plays an important role in apoptosis and be targeted by TAGE in GA-treated HepG2 cells. Caspase-3 was shown to be cleaved and activated during apoptosis, resulting in protease activity [88]. However, as TAGE-modified caspase-3 levels increased, reductions were noted in protease activity and the cleavage of poly (ADP-ribose) polymerase, which is downstream of caspase-3 in the apoptotic cascade. A previous study reported that necrotic-like cell death was enhanced by elevations in TAGE-modified caspase-3 levels [10]. Although the relationship between TAGE-modified caspase-3 and necrosis-like cell death currently remains unknown, TAGE-modified caspase-3 may play an important role in the mechanisms underlying cell death in the pathogenesis of NASH [85].

### 6.3. Oxidative Stress and Its Response Associated with Intracellular TAGE in Hepatocytes

Although hepatocyte cell death is a common feature of NASH, the contributing factors and the mechanisms underlying its pathogenesis have yet to be clarified. TAGE-induced protein dysfunction is expected to suppress stress defense mechanisms in cells, which may easily lead to cell death. Further studies on the mechanisms responsible for TAGE-induced cell death are needed to elucidate its relevance to NASH.

Oxidative stress is a typical factor of stress induced in cells, and its involvement in the pathogenesis of disease has been reported in relation to NASH [89]. HepG2 cell death correlated to the intracellular accumulation of TAGE was recently shown to be inhibited by N-acetyl-L-cysteine, an antioxidant [11]. Furthermore, increases in ROS levels were observed in GA-treated HepG2 cells and human primary hepatocytes, suggesting the involvement of accumulated TAGE in intracellular oxidative stress and ROS-induced cell death [11].

Intracellular oxidative stress may be attributed to the collapse of oxidative defense mechanisms or an increase in ROS generation. Antioxidant defense mechanisms in cells include the transcriptional factor nuclear factor erythroid 2-related factor 2 (Nrf2), which activates a number of detoxification enzymes [90]. Nrf2 plays an important role in NAFLD and NASH. A previous study demonstrated that the onset and progression of NASH were rapid in mice with a Nrf2 deficiency [91]. Furthermore, NASH patients with lower glutathione levels exhibited the higher expression of hemeoxygenase-1 (HO-1), which are downstream of Nrf2 [92]. In addition, catalase activity was increased in the livers of mice fed a high-Fru plus high-fat diet, which was expected to result in the accumulation of TAGE, and these mice also showed fat accumulation and increased inflammation [93]. Furthermore, the expression of Nrf2 and HO-1 was up-regulated and catalase activity was maintained in GA-treated HepG2 cells [11]. On the other hand, mitochondrial abnormalities have been shown to enhance intracellular oxidative stress under conditions of increased ROS generation [89]. An increase in ROS correlated with mitochondrial abnormalities in HepG2 cells treated with GA [11]. The multiple parallel hits theory has been proposed as a cause of NASH progression. In the case of TAGE accumulation, which has been suggested as one of the causes of NASH progression, the main phenotype is predicted to be an increase in oxidative stress due to mitochondrial abnormalities that cause cytotoxicity, rather than a reduction in antioxidant function as the pathogenic pathway.

A relationship has been reported between the production of ROS and inflammation. A previous study demonstrated that ROS up-regulated the expression of C-reactive protein (CRP) in the human normal liver cell line, L-02 [94]. Furthermore, the intracellular accumulation of TAGE in Hep3B cells was associated with the up-regulated expression of CRP mRNA [9]. Inflammatory responses associated with TAGE accumulation have been detected not only in HCC cells, but also in hiPSC-HLC [12]. Furthermore, the accumulation of TAGE in hiPSC-HLC correlated with the up-regulated expression of inflammation-related genes, including monocyte chemoattractant protein-1 (MCP-1) and interleukins 6 and 8 [12]. Experimental data from hiPSC-HLC as a model of human liver cells derived from non-cancer cells more accurately reflect the disease pathology of humans. The correlation between TAGE accumulation and inflammation in the HCC cell lines HepG2/Hep3B and hiPSC-HLC implicates TAGE accumulation in the liver as a cause of inflammatory NASH.

Collectively, these findings indicate that increases in intracellular ROS production with the accumulation of TAGE cause inflammation and, ultimately, cell death, and provide novel insights into the contribution of TAGE to the onset and progression of NASH.

### 6.4. Effects of Extracellular TAGE on Hepatocytes

Extracellular TAGE are also involved in inflammatory responses in hepatocytes. The TAGE–RAGE axis promotes inflammation in hepatocytes. A previous study reported increases in the expression levels of CRP in TAGE-treated Hep3B cells [70]. Furthermore, Rac-1 mediated the expression of CRP by activating NF-κB or NOX [95], the expression of which was high in NASH patients/mice [96,97]. NOX is expected to generate ROS and enhance inflammation in Hep3B cells.

TAGE–RAGE signaling in Hep3B cells has been detected in EC. EC dysfunction has been implicated in the progression of NAFLD [98]. NOX-induced ROS generation via the TAGE–RAGE axis was previously shown to be mediated by the activation of the transcriptional factors NF-κB and AP-1 [68,99,100]. The TAGE–RAGE axis was also activated by inflammatory and thrombogenic reactions in EC via the expression of plasminogen activator inhibitor-1, intercellular adhesion molecule-1 (ICAM-1), and MCP-1 through the production of ROS [24,101,102,103].

These findings suggest that TAGE induces inflammation through its extracellular and intracellular effects and contributes not only to the onset, but also the progression of NAFLD/NASH.

### 6.5. Effects of Extracellular TAGE on HSC

In CLD, including NASH, the pathological accumulation of extracellular matrix (ECM) proteins leads to liver fibrosis, the progression of which induces cirrhosis [104]. In CLD, HSC are activated by various cytokines, including transforming growth factor-β1 (TGF-β1), platelet-derived growth factors, and TNF-α, and differentiate into myofibroblast-like cells, which then secrete high levels of ECM material, such as collagen type I (COL-I) [105].

Intracellular oxidative stress was previously induced in TAGE-treated LI90 cells, a human HSC line, through the production of RAGE-NOX-derived ROS [106]. Furthermore, the up-regulated expression of the genes and proteins that play roles in fibroblastogenesis and inflammation, including α-smooth muscle actin, COL-Iα2, TGF-β1, and MCP-1, was observed in these cells. Therefore, TAGE appear to be involved in the onset and progression of liver fibrosis by promoting ROS production and HSC activation via RAGE.

TGF-β1-induced apoptosis in the human HSC line LX-2 was inhibited by TAGE. In addition, the culture medium of LX-2 cells co-treated with TGF-β1 and TAGE showed significantly elevated levels of the COL-I protein, whereas COL-I expression levels remained unchanged [107]. Based on these findings, TAGE promoted the generation of ECM molecules, such as COL-I, through the suppression of apoptosis in TGF-β1-activated HSC.

These findings suggest that TAGE worsens liver fibrosis in chronic hepatitis patients, such as those with NASH.

### 6.6. Extracellular TAGE and the Malignant Progression of NASH-Related HCC

The progression of NASH is associated with an increased risk of the onset of cirrhosis and HCC. The TAGE–RAGE axis has been suggested to play a role in the malignant progression of NASH-related HCC. Elevated CRP levels predict a poor clinical outcome in HCC patients. TAGE were previously found to increase CRP expression levels in HCC, and this was prevented by a pretreatment with anti-RAGE antiserum. Moreover, the risk of the malignant transformation of HCC was reduced by sRAGE, a decoy receptor of RAGE. These findings indicate the potential of the TAGE–RAGE axis as a therapeutic target for NASH-related HCC [70].

In addition to CRP, VEGF expression levels were previously shown to be elevated in HCC [108]. TAGE up-regulated the expression of VEGF in Hep3B cells [69]. Furthermore, proteins secreted by TAGE-treated Hep3B cells promoted the angiogenesis of EC, which is important for tumorigenesis, indicating that the TAGE–RAGE signaling pathway increased the angiogenic potential of HCC cells by up-regulating the expression of VEGF [69]. A relationship has been reported between TAGE and VEGF not only in hepatocytes, but also in vivo. A high-AGE beverage orally administered to rats induced the hepatic expression of VEGF and accumulation of Glu-AGEs, which predicted increases in TAGE due to abnormalities in the intracellular metabolism of Glu [24,109]. These findings suggest that dietary AGEs play a pathological role in the progression of HCC.

We previously demonstrated that the expression of VEGF was up-regulated by NOX-induced ROS production via the TAGE–RAGE axis through the transcriptional activation of NF-κB and AP-1, which, in turn, increased the proliferation of EC and tube formation, key factors in tumor angiogenesis [69,99,110,111]. Furthermore, inflammatory and thrombogenic reactions were induced in EC by the activation of the TAGE–RAGE axis through the up-regulated expression of MCP-1 and ICAM-1 via ROS [101,102,103,112].

Therefore, TAGE appear to play a role in the proliferation and invasion of cancer cells through RAGE interactions.

### 6.7. Limitations

TAGE stress in NASH is being investigated both in vivo and in vitro, including clinical specimens, animal models, and cells. Insights have been obtained into the relationship between TAGE stress and oxidative stress, particularly from studies using hepatocytes, and further research on TAGE target molecules is expected in the future. AGEs and ROS are closely related, and relationships have been reported for other AGEs and diseases [113,114]. While the findings obtained on other AGEs are important, this review focused on TAGE, which is relevant to NASH.

The majority of studies conducted on AGEs to date have examined the effects of the AGE-RAGE axis from outside of the cell, and thus, a more detailed understanding of the cytotoxic effects of intracellular AGEs is important. Further studies on the effects of TAGE produced by Glu/Fru metabolism are needed.

## 7. Conclusions and Perspectives

**Figure 2:**
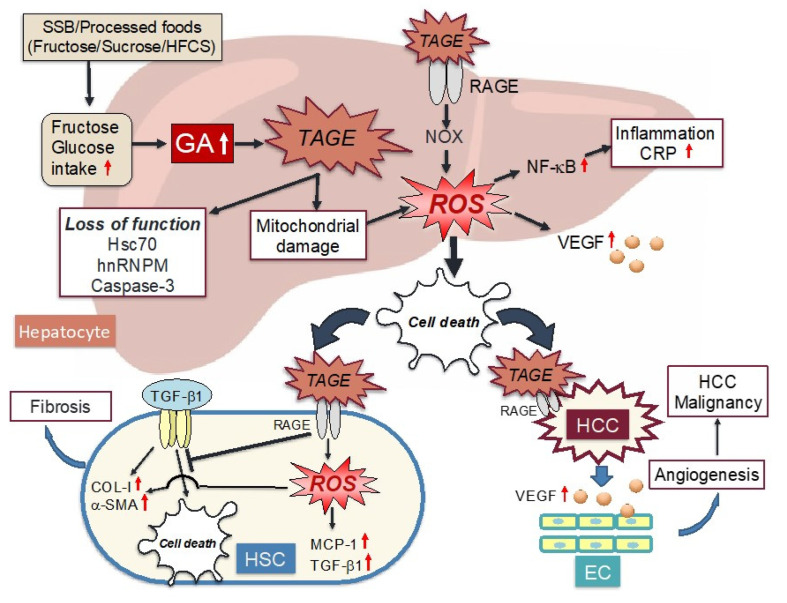
Involvement of intracellular TAGE and the TAGE–RAGE–ROS axis in the onset and progression of NAFLD/NASH.

SSB and/or processed foods in the modern daily diet, when consumed repeatedly and at excessive amounts, elevate cellular levels of GA and promote TAGE modifications to intracellular proteins. The accumulation of intracellular TAGE has been associated with cell death, which may contribute to the leakage of TAGE into extracellular regions and increases in serum TAGE levels. Extracellular TAGE contribute to not only the onset, but also the progression of NAFLD/NASH through their activation of the TAGE–RAGE axis, which enhances ROS generation and increases RAGE expression levels (Figure 2). The “TAGE theory” provides novel insights for future studies on NAFLD/NASH and HCC.

The chronic intake of excessive amounts of SSB and processed foods increases cellular levels of the sugar metabolite GA, which, in turn, enhances TAGE production. TAGE accumulation is associated with the loss of protein function and mitochondrial membrane abnormalities. Damage to mitochondria results in the intracellular production of ROS, which ultimately leads to hepatocyte death. TAGE-modified proteins that accumulate intracellularly are expected to leak out of cells upon cell death and exert various effects on the surrounding cells via the TAGE–RAGE axis. The TAGE–RAGE axis promotes the production of ROS by NOX. TAGE-induced ROS production may be one cause of oxidative stress in NASH. Redox-sensitive transcription factors, including NF-κB, may be activated by ROS, which, in turn, promotes the production of cytotoxic, pro-inflammatory, and fibrogenic mediators by hepatocytes and/or HSC, thereby contributing to the progression of NAFLD/NASH. TAGE also suppress TGF-β1-induced HSC cell death and maintain HSC. Therefore, the increased production of ECM molecules and eventual fibrosis are expected. The interaction between TAGE and RAGE affects intracellular signaling in tumor cells and HSC, and induces angiogenesis, invasion, migration, proliferation, and fibrosis. This cooperation by the TAGE–RAGE axis may lead to the malignant progression of NASH-related HCC. SSB: sugar-sweetened beverages; HFCS: high-fructose corn syrup; GA: glyceraldehyde; TAGE: toxic AGEs; RAGE: receptor for AGEs; NOX: nicotinamide adenine dinucleotide phosphate reduced oxidase; ROS: reactive oxygen species; Hsc70: heat shock cognate 70; hnRNPM: heterogenous nuclear ribonucleoprotein M; NF-κB: nuclear factor kappa B; VEGF: vascular endothelial growth factor; CRP: C-reactive protein; HSC: hepatic stellate cells; TGF-β1: transforming growth factor-β1; MCP-1: monocyte chemoattractant protein-1; COL-I: collagen-type I; α-SMA: α-smooth muscle actin; HCC: hepatocellular carcinoma; EC: endothelial cell.

## Data Availability

Not applicable.

## Abbreviations

The following abbreviations are used in this manuscript:

AGEs	Advanced glycation end-products
CLD	Chronic liver disease
CML	N^ε^-(Carboxymethyl)lysine
COL-I	Collagen type I
CRP	C-Reactive protein
CVD	Cardiovascular disease
3-DG-AGEs	3-Deoxyglucosone-derived AGEs
DHAP	Dihydroxyacetone phosphate
DN-HD	Diabetic nephropathy with hemodialysis
EC	Endothelial cells
ECM	Extracellular matrix
Fru	Fructose
Fru-AGEs	Fructose-derived AGEs
GA	Glyceraldehyde
GA-AGEs	Glyceraldehyde-derived AGEs
GA-3-P	Glyceraldehyde-3-phosphate
GAPDH	Glyceraldehyde-3-phosphate dehydrogenase
GLAP	3-Hydroxy-5-hydroxymethyl-pyridinium compound
Glu	Glucose
Glu-AGEs	Glucose-derived AGEs
Glycol-AGEs	Glycolaldehyde-derived AGEs
GO	Glyoxal
GO-AGEs	Glyoxal-derived AGEs
γ-GTP	γ-Glutamyl transpeptidase
HCC	Hepatocellular carcinoma
HFCS	High-fructose corn syrup
hiPSC	Human induced pluripotent stem cells
hiPSC-HLC	HLC differentiated from hiPSC
HLC	Hepatocyte-like cells
hnRNPM	Heterogeneous nuclear ribonucleoprotein M
HO-1	Hemeoxygenase-1
HOMA-IR	Homeostasis model assessment of IR
Hsc70	Heat shock cognate 70
HSC	Hepatic stellate cells
ICAM-1	Intercellular adhesion molecule-1
IL	Interleukin
IR	Insulin resistance
MCP-1	Monocyte chemoattractant protein-1
MG-H1	Methylglyoxal-derived hydroimidazolone compound
MGO	Methylglyoxal
MGO-AGEs	Methylglyoxal-derived AGEs
MS	Metabolic syndrome
NAFL	Nonalcoholic fatty liver
NAFLD	Nonalcoholic fatty liver disease
NASH	Nonalcoholic steatohepatitis
NBNC-HCC	Non-B, non-C HCC
NF-κB	Nuclear factor kappa B
NOX	Nicotinamide adenine dinucleotide phosphate-reduced oxidase
Nrf2	Nuclear factor erythroid 2-related factor 2
RAGE	Receptor for AGEs
sRAGE	Soluble form of RAGE
RasGRP2	Ras guanyl nucleotide releasing protein 2
ROS	Reactive oxygen species
α-SMA	α-smooth muscle actin
SSB	Sugar-sweetened beverages
TAGE	Toxic AGEs
T2DM	Type 2 diabetes mellitus
TGF-β1	Transforming growth factor-β1
TNF-α	Tumor necrosis factor-α
VE	Vascular endothelial
VEGF	Vascular endothelial growth factor
